# Exposing the potential of XAI-based causal discovery for analysing unstable rock slopes

**DOI:** 10.1038/s41598-026-48268-x

**Published:** 2026-04-24

**Authors:** Lukas Schild, Thomas Scheiber, Paula Snook, Alexander Maschler, Reza Arghandeh

**Affiliations:** 1 Department of Civil Engineering and Environmental Sciences, Western Norway University of Applied Sciences, Sogndal, Norway; 2 Department of Computer science, Electrical engineering and Mathematical sciences, Western Norway University of Applied Sciences, Bergen, Norway

**Keywords:** Natural hazards, Solid Earth sciences

## Abstract

Unstable rock slopes pose significant challenges in geohazard analysis due to their complex nature. Traditional approaches to understanding displacement drivers on unstable rock slopes limit conclusive statements on process drivers, focusing on statistical analyses and expert knowledge. eXplainable Artificial Intelligence (XAI) based Causal Discovery enables the unbiased construction of causal graphs, depicting the cause-and-effect relations between system variables, beyond statistical correlations. In this study, we use Multi-Window Causal Discovery (MWCD) for creating causal graphs from pre-failure displacement and weather monitoring data applied to three different sites. At the Stampa rock section 4a (Norway), MWCD identifies a temporal shift from an indirect to a direct effect of precipitation and infiltration on the displacement, corresponding to a final destabilization. In Preonzo (Switzerland), causal connections change considerably during the pre-failure acceleration from temperature and precipitation dominated effects to a gravitationally driven system. At Veslemannen (Norway), MWCD identifies spatial causality with lower sections of the instability strongly influencing the deformation dynamics of the upper sections. Our results show that incorporating XAI-based methods alongside traditional geological analysis drastically improves the understanding of displacement dynamics in unstable rock slopes. This improved understanding can be used to build more robust and accurate forecasting methods.

## Introduction

Unstable rock slopes that potentially fail catastrophically pose significant threats to communities and infrastructure. Hence, comprehensive risk management necessitates a thorough understanding of the factors driving slope deformations. The failure of rock slopes is usually a consequence of long-term preparatory factors, e.g. hydrostatic pressure and weathering^1^. Moreover, exogenous variables such as precipitation and temperature, which can be monitored using standard meteorological stations, have received particular attention as drivers of short-term displacement^2^. For example, infiltrating water from precipitation or snowmelt can destabilise rock slopes through increased hydrostatic pressure in fractures and warm temperatures may lead to the degradation of permafrost and thus enhance slope deformation^3–5^. Traditional approaches to understanding the drivers of rock slope failures include expert knowledge and statistical analysis, which relies on observed coincidences of events such as heavy precipitation and catastrophic failures or correlation of pre-failure displacement and meteorological conditions^6–8^, e.g.. In addition, simulation techniques are employed to reproduce the observed displacement, for example, based on hydro-mechanical properties of the rock^9^. These traditional methods generally limit conclusive statements based on observational data regarding the process drivers due to their focus on statistical correlation. This focus impedes a comprehensive understanding and assertions of the underlying causal relations^10^. Furthermore, simulation based approaches including Limit Equilibrium and Finite Element methods focus on slope internal properties such as fracture propagation and disregard the role of external drivers^11–13^, e.g.. Causal Discovery approaches are advanced methods that aim at developing a Structural Causal Model (SCM), establishing cause-and-effect relations between system variables. Based on the theory of Causality, a Directed Acyclic Graph (DAG) represents the cause and effect relations along directed edges with the system variables as nodes^14^. Approaches to Causal Discovery include constraint-based methods such as the PC algorithm and its variants^15–18^, aiming at constructing a SCM based on statistical independence tests. Score-based approaches, such as Greedy Equivalence Search (GES)^19^, DAGs with NO TEARS^20^, NoTearsNonLinear^21^ and its improved deep learning based version DAG Structure Learning with Graph Neural Networks (GNNs)^22^ construct the causal graph based on optimising a score criterion. Asymmetry-based methods such as Linear Non-Gaussian Acyclic Model Causal Discovery (LiNGAM)^23^ and its direct entropy-based extension DirectLiNGAM^24^ discover the underlying causal structure by leveraging asymmetries in the data distributions.

Within the field of geoscience, Causal Discovery already finds application in a variety of domains, such as exploring the effect of environmental variables on land surface changes^25^, inspecting the drivers for irrigation water use^26^, and modelling the interaction of large scale weather systems^27^. In the context of unstable rock slopes, applying Causal Discovery methods have yet to be explored to its full potential.

**Fig. 1:**
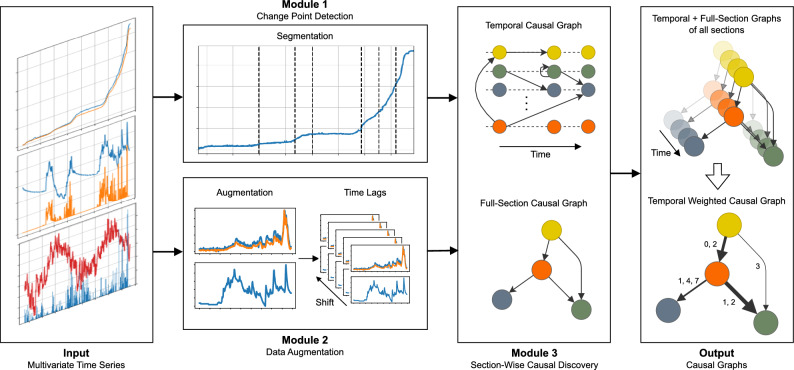
Causal Discovery framework using the MWCD algorithm. Surface displacement and environmental data from a monitoring setup serve as input data. **Module 1** computes the change points in the target time series to split the time series for all variables into stationary time windows. The data is augmented in **Module 2** by additional time series derived from the original data. This typically includes data such as negative-degree days, FTCs and consecutive negative-degree days or FTC days. Furthermore, lagged versions of the time series are created for each window. The results of **Module 1** and **2** are used in **Module 3** as input to a Causal Discovery algorithm, where the colours indicate different input variables. To compute the final output graph, all causal graphs are averaged. The resulting graph edge thickness represents the average occurrence rate of the edge in all graphs, while the edge label indicates the time lag in number of samples between cause and effect. Combined, the temporal (lagged) and weighted graph conveys the Causal Discovery result. All windows can be viewed individually to analyse changes in the causal graphs over time.

In this paper, we explore driver analyses based on Causal Discovery using eXplainable Artificial Intelligence (XAI) aiming at establishing causal relations between variables from observational data^18^. Specifically, we propose Causal Discovery using GNNs. Compared to more traditional methods relying predominantly on expert knowledge, machine learning-based methods exhibit less cognitive bias due to their data-driven nature and impose only few assumptions, such as stationarity, on the input data^28^. This reliance on observations rather than educated guesses and domain knowledge for the initial causal discovery makes XAI based Causal Discovery well-suited for complex geological processes^27^. Moreover, with advances in computation, training neural networks has become more affordable, making XAI-based methods increasingly popular^29^. To overcome one limitation of Causal Discovery imposed by the assumption of a stationary process, we propose the application of the Multi-Window Causal Discovery (MWCD) algorithm (Fig. 1). The inherently non-stationary displacement data is split into stationary time windows, in which both the exogenous and endogenous variables are stationary. The differentiated displacement in the time windows is then used to perform Causal Discovery. The result is a Causal Graph that combines temporal information with uncertainty on causal links in the graph. The causal graphs from different windows can be compared to extract information on the temporal evolution of the underlying processes and the interplay of driving factors. We believe that Causal Discovery approaches can significantly advance our understanding of unstable rock slope drivers, and we demonstrate their exploration and application in this context.

## Results

We tested the potential of the MWCD approach on three different datasets from unstable rock slope sites in Europe. The sites differ in location (latitude), geological setting (bedrock structure and rock types) and evolution of displacement patterns over time but are comparable with respect to displacement drivers, thus forming the basis for a comparative analysis. The datasets from the three sites differ in available types of data, but they always include directly measured displacement data, as well as measured and modelled environmental data. However, data availability regarding environmental variables differs between the sites and is provided in the tables below. All the datasets were augmented to include information on negative-degree days and diurnal Freeze-Thaw-Cycles (FTCs). The augmented data is added to conserve information on absolute values that would be lost during scaling but are assumed to constitute a causal factor^7^. On one hand, one fundamental challenge concerning the evaluation of the generated causal models is the lack of reference models for real-world datasets. This challenge is particularly relevant for unstable rock slopes, where the true causal graph of the system is unknown and cannot be constructed with certainty from domain knowledge. It is therefore not possible to quantitatively validate the results. However, known interactions between environmental variables, such as precipitation and infiltration, can be used as an indicator for the validity of a causal graph. On the other hand, this challenge demonstrates the great potential of XAI-based Causal Discovery in geosciences, allowing to generate information which cannot be obtained through traditional research methods and is particularly strong in combination with traditional methods and expert knowledge. To minimise any restriction or bias on the method, the MWCD does not require expert input to perform Causal Discovery.

In the following, the three unstable rock slopes characterised by different spatial extents, climatic and geological settings as well as different deformation patterns are analysed using MWCD. In each case, causal graphs are used to explain the influence of environmental factors on observed surface displacement. The collection of window-wise graphs provides a visual representation of the system’s evolution over time, including the changing causal connections and time lags between cause and effect. The case studies illustrate the application of the MWCD and the analysis of the resulting causal graphs.

### Weighted and temporal causality at Rock Section 4a, Stampa, Norway

**Fig. 2:**
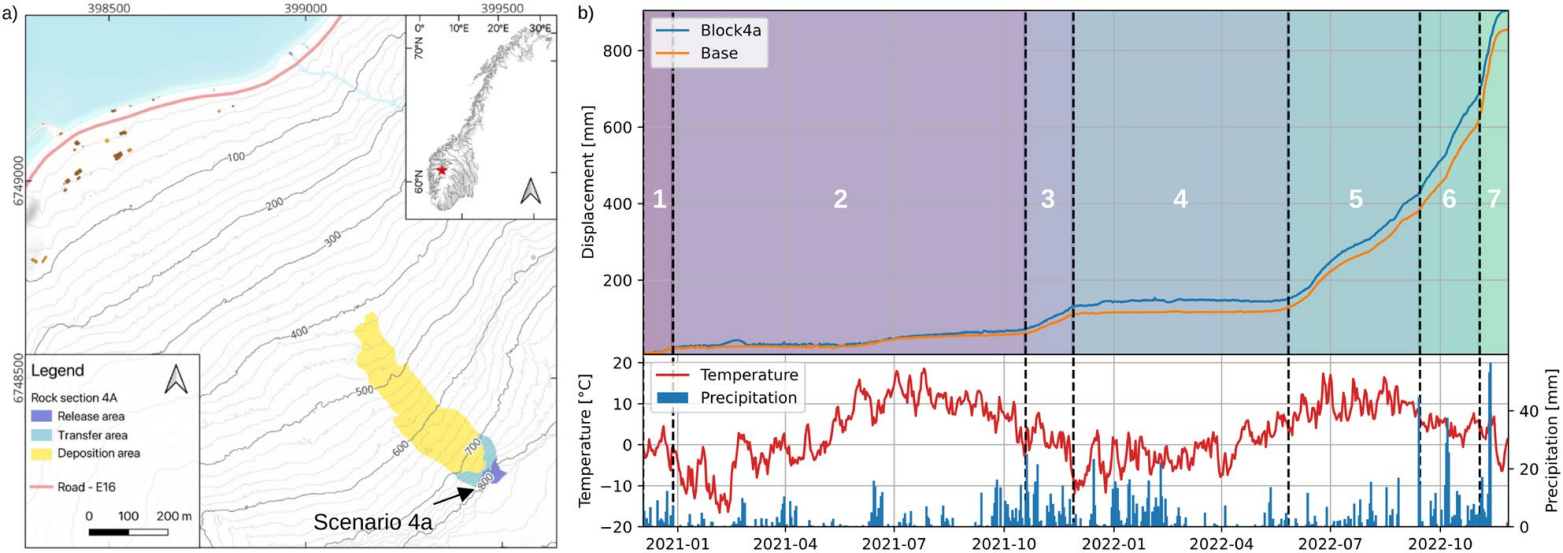
**a**) Geographical overview of the Stampa Study Site. **b**) Displacement time series for *Block 4a* and the *Base* (top) and observed daily mean air temperature and daily precipitation (bottom). The dotted lines indicate the computed change points that split the time series into time windows. Map data provided by Kartverket (www.kartverket.no), map generated in QGIS 3.34 (www.qgis.org).

Rock Section 4a was approximately $54,000 \ m^3$ in volume and part of the unstable rock slope Stampa located in western Norway (Fig. 2a). The rock section collapsed in July 2023 as a two-stage failure including an upper part, a rock column named *Block 4a* with an approximate volume of $5,000 \ m^3$ and a *Base* with a volume of $49,000 \ m^3$. The displacement data used in this study stem from an on-site sensor network and remote sensing and cover the period from November 2020 until December 2022, with a considerable acceleration in the monitored displacement from June to December 2022 (Fig. 2b). Oppikofer et al.^30^ and Schild et al.^31^ provide a more detailed description of the study site and the data from the on-site sensor network, respectively. Besides displacement of *Block 4a* and its *Base*, the MWCD input data consists of daily mean air temperature and accumulated precipitation recorded by an on-site weather station and soil moisture and infiltration accessed via Varsom Xgeo (www.xgeo.no), an open-access decision-support tool (Tab. 1).

**Table 1:** Input data used for a causal analysis at Rock Section 4a. All data have been resampled to a resolution of 1 day before applying MWCD.

**Data type**	**Source (instrument or model)**	**Temporal resolution**
Displacement Block 4a	Robotic Total Station,	2 hours
	Satellite-based InSAR	6 days
	Ground-based InSAR	24 hours
	and Extensometer^31^	1 hour
Displacement Base	Robotic Total Station,	2 hours
	Satellite-based InSAR	6 days
	Ground-based InSAR	24 hours
	and Extensometer^31^	1 hour
Daily Mean Air Temperature	On-site Weather Station	1 day
Daily Accumulated Precipitation	On-site Weather Station	1 day
Soil Moisture	HBV Model^32^	1 day
Infiltration	HBV Model^32^	1 day
Freeze-Thaw Cycle Days	Derived from *Daily Mean Air Temperature*	1 day
Negative Degree Days	Derived from *Daily Mean Air Temperature*	1 day

Soil moisture and infiltration are simulated based on an interpolated precipitation map and a grid based version of the Hydrologiska Byråns Vattenbalansavdelning (HBV) hydrology model, using a 30-year reference period (1981-2010). The HBV model simulates evapotranspiration, infiltration and run-off, enabling the estimation of infiltration and soil moisture^33,32^. Despite the lack of better data and based on the fact that the unstable rock slope experienced water infiltration into rock fractures, we believe that infiltration and soil moisture are important proxies to include.

**Fig. 3:**
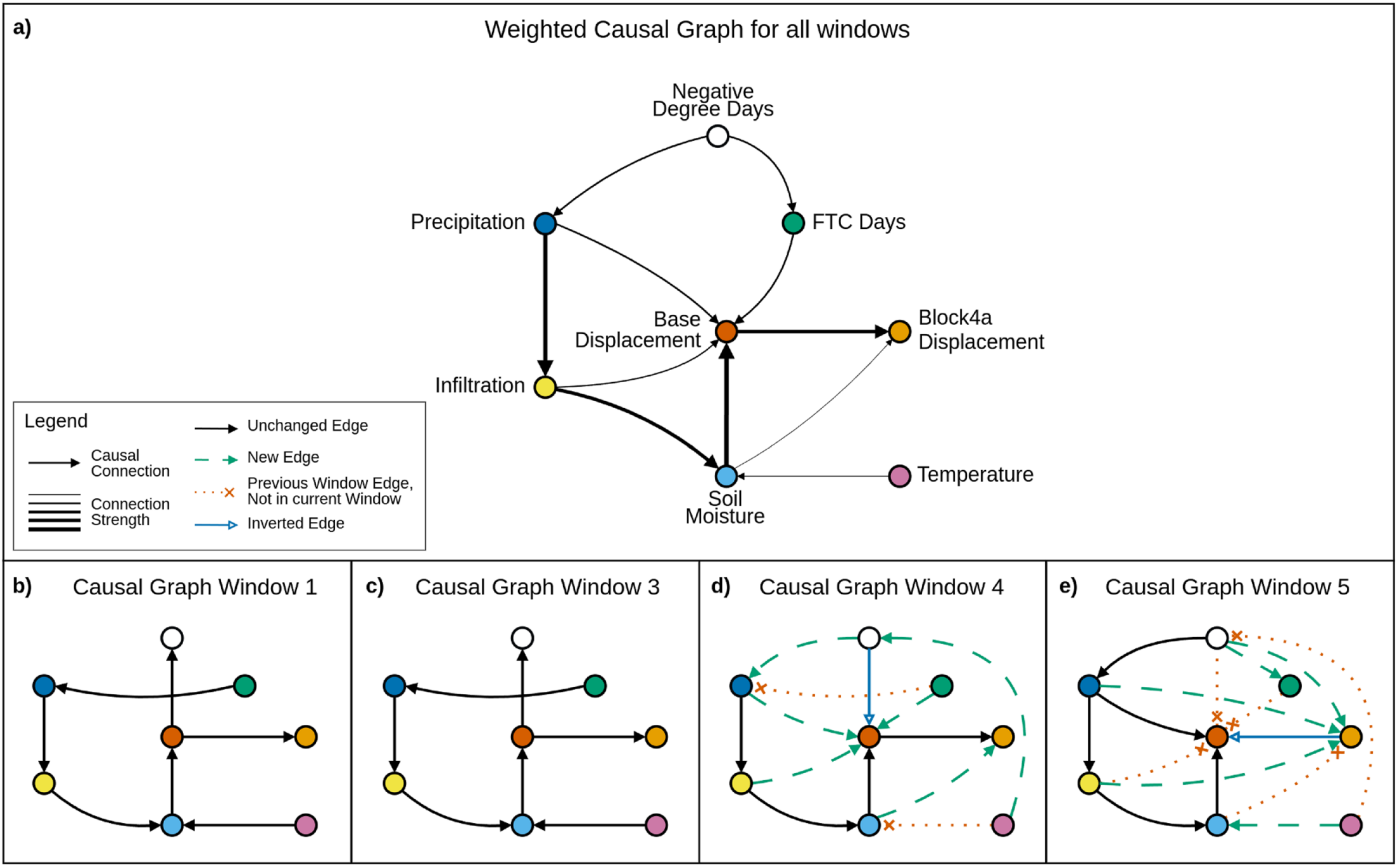
Overview of the results using MWCD for Causal Discovery on the rock section 4a dataset. a) Weighted causal graph that condenses the information over all time windows (Fig. 2b). The arrow indicates the direction of causation and the line thickness indicates how often an edge occurred throughout the time windows. b)-e) Causal graphs for the respective time window illustrating the temporal evolution of the causal connections.

The Change Point Detection (CPD) splits the dataset into seven time windows based on shifts in the displacement velocities (Fig. 2b). The results show that MWCD is a powerful tool to identify causality between different environmental factors that drive the stability and displacement of unstable rock sections: At this site, soil moisture significantly influences the displacement of the *Base*, but less so the movement of *Block 4a* (Fig. 3a). The displacement of *Block 4a* is mostly driven by the displacement of the *Base* (Fig. 3a). Precipitation, infiltration and consecutive FTC days, indicating the transition from frozen ground conditions to thawing conditions, directly affect the displacement of the *Base* rock section. However, precipitation and infiltration have the strongest influence on displacement indirectly through soil moisture. The latter is highlighted by the high occurrence rates of the causal edges between these variables (connection strength). Expert assessments based on basic data analysis and in-situ observations previously suggested that high water availability through rain and snowmelt likely increased hydrostatic pressure at depth, leading to enhanced displacement of the rock slope. This is a known destabilising factor at depth, verified by laboratory tests and physical modelling^4^. However, in this case, traditional statistical analyses did not reveal significant correlations between water availability and displacement. Our causality analysis therefore represents the only data-based possibility to corroborate these assumptions.

Moreover, the MWCD provides insights into the temporal evolution of the system, identifying a shift from an indirect (Fig. 3b,c) to a direct (Fig. 3d,e) effect of precipitation and infiltration on the displacement. This causal relation change indicates that gradual destabilisation led to a more immediate reaction of the rock section to precipitation. This finding underlines the potential of MWCD for long-term analysis and anomaly detection.

### Temporal and spatial causality at Preonzo, Switzerland

**Fig. 4:**
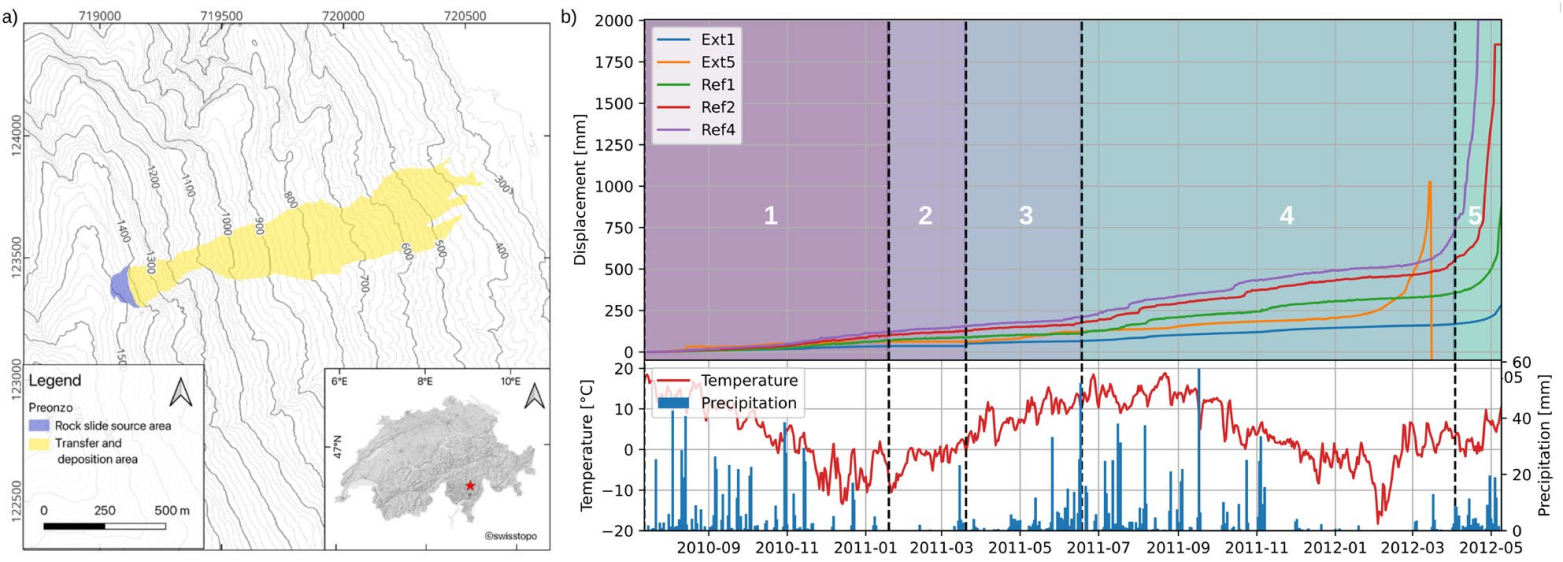
**a**) Overview map of location and failure at the Preonzo site (modified from Loew et al.^34^). **b**) Displacement and weather data time series for two extensometers, three geodetic reflectors and mean temperature as well as precipitation. The dashed lines indicate the time windows as computed by the CPD algorithm. Map data provided by Swisstopo (www.map.geo.admin.ch), map generated in QGIS 3.34 (www.qgis.org).

The second dataset originates from a complex instability at Alpe di Roscioro, near the village of Preonzo in southern Switzerland (Fig. 4a). The rock slope failed catastrophically on May 15th 2012, with a volume of approximately $210,000 \ m^3$^34^. The Causal Discovery of the driving factors is based on displacement data from Leinauer et al.^35^ as well as weather data and ground conditions obtained through the OpenMeteo weather API^36^. The latter consists of a model-based approximation of soil moisture and ground temperature data at different depths ($0-7 \ cm$, $7-28 \ cm$, $28-100 \ cm$ and $100-255 \ cm$) as well as daily mean air temperature and precipitation. From the in-situ monitoring setup, we selected two extensometers (Ext1 and Ext5) and three geodetic reflectors (Ref1, Ref2 and Ref4) for the Causal Discovery since the spatial distribution of those displacement measures allows for identifying slope dynamics across different parts of the unstable slope (Tab. 2).

**Table 2:** Input data used for a causal analysis at Preonzo. All data have been resampled to a resolution of 1 day before applying MWCD.

**Data type**	**Source (instrument or model)**	**Temporal resolution**
Displacement Ext1	Extensometer^35^	1 hour
Displacement Ext5	Extensometer^35^	1 hour
Displacement Ref1	Robotic Totalstation^35^	20 min
Displacement Ref2	Robotic Totalstation^35^	20 min
Displacement Ref4	Robotic Totalstation^35^	20 min
Ground Temperature	Model Based Approximation^36^	1 day
Soil Moisture	Model Based Approximation^36^	1 day
Daily Mean Air Temperature	Model Based Approximation^36^	1 day
Daily Accumulated Precipitation	Model Based Approximation^36^	1 day
Daily Mean Snowfall	Model Based Approximation^36^	1 day
Freeze-Thaw Cycle Days	Derived from *Daily Mean Air Temperature*	1 day
Negative Degree Days	Derived from *Daily Mean Air Temperature*	1 day

CPD was performed on the displacement velocities obtained from extensometer 1, resulting in five time windows for the MWCD (Fig. 4b).

**Fig. 5:**
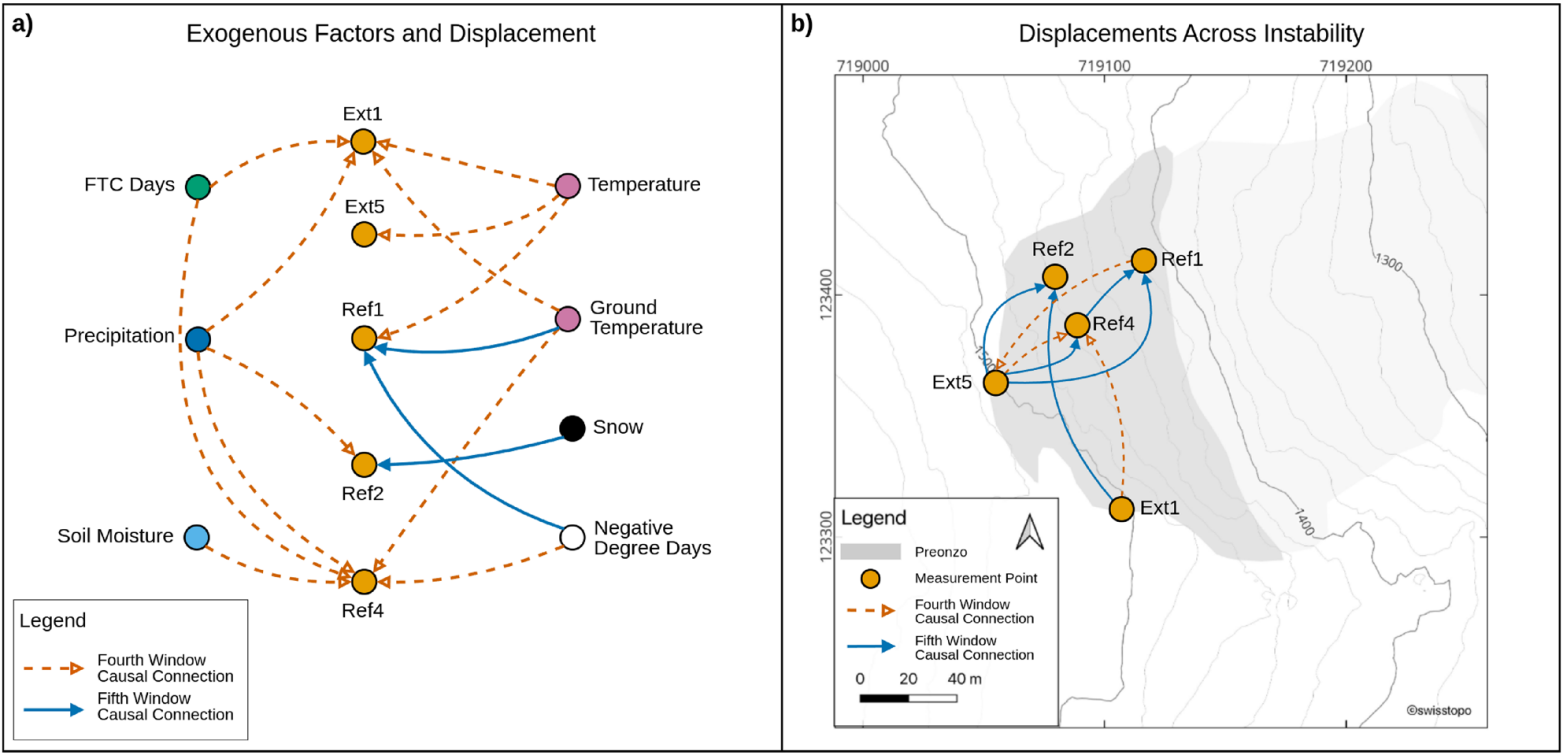
Changes of exogenous factors and displacement from window four to window five for the Preonzo case study site. **a**) Changing causal connections between exogenous factors and displacement in the fourth and fifth time window (Fig. 4b). **b**) Changing causal connections for the different sections of the slope between the fourth and fifth time window (Fig. 4b). Map data provided by Swisstopo (www.map.geo.admin.ch), map generated in QGIS 3.34 (www.qgis.org).

The causal connections change considerably with time, as illustrated by the causal graphs from time windows four and five (Figs. 4b and 5a). The fourth time window is dominated by connections from precipitation, air temperature and FTCs to displacement, while displacement in the fifth window is, according to the MWCD, mostly influenced by the external variables ground temperature and negative degree days (Fig. 5a). At the same time, the MWCD results illustrate that the interactions between different parts of the slope change to a dominant movement on the upper part in time window five that drives the displacement on the lower part of the slope (Fig. 5b). These results can be interpreted as a shift in driving forces from external variables to internal stress distribution and thus signify a transition to a predominantly gravitational force-driven system towards failure. This finding is in accordance with Loew et al.^34^, who suggested hydromechanical loading as the actual driving factor of displacement acceleration. Thereby, our MWCD-based analysis aids in identifying crucial short-term system changes, which may be associated with imminent rock-slope failures.

### Spatial causality at Veslemannen, Norway

**Fig. 6:**
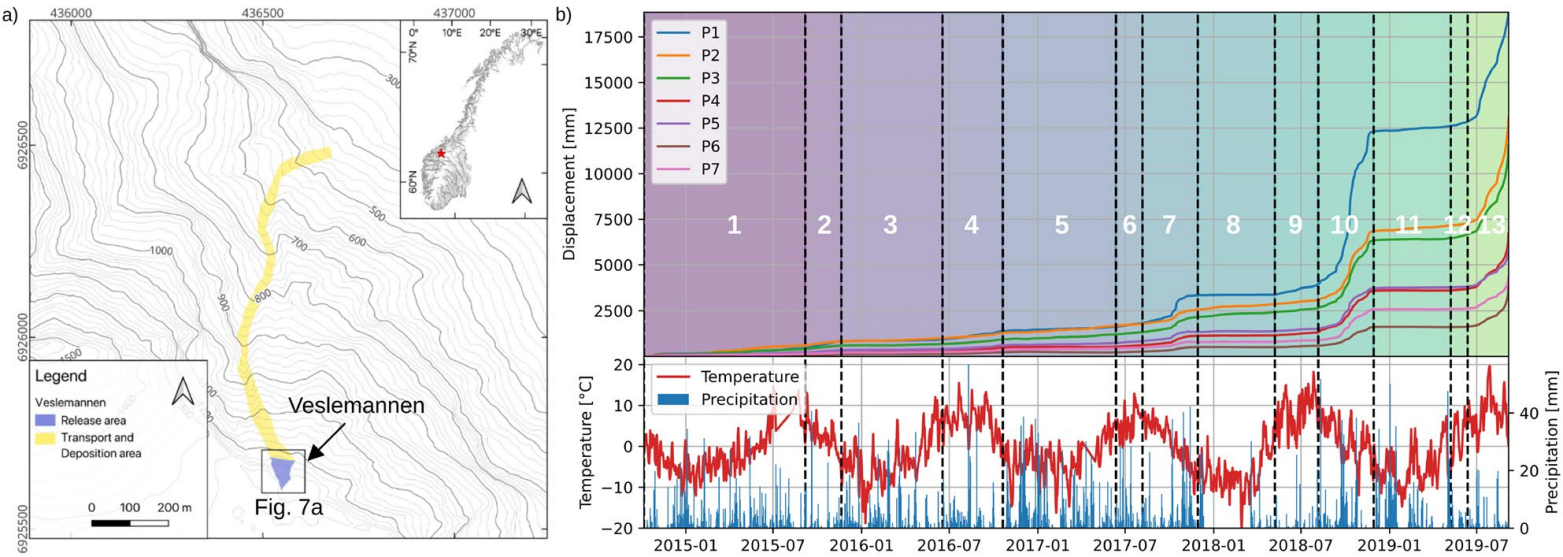
**a**) Map showing the location of the Veslemannen study site (extent of rockslide after^6^). **b**) Displacement and weather data time series for the seven GB-InSAR points, ’P1’ to ’P7’, daily mean temperature and precipitation. The dashed lines indicate the windows as computed by the CPD algorithm applied to the displacement data. Map data provided by Kartverket (www.kartverket.no), map generated in QGIS 3.34 (www.qgis.org).

Veslemannen was part of a larger rock slope instability called Mannen (Fig. 6a) and failed on September 5th, 2019, with a volume of approximately $54,000 \ m^3$. We use displacement data from seven ground-based radar interferometry (GB-InSAR) points covering different sections of Veslemannen and combined them with weather data from Varsom Xgeo (www.xgeo.no) as MWCD input.

**Table 3:** Input data used for a causal analysis at Veslemannen. All data have been resmampled to a resolution of 1 day before applying MWCD.

**Data type**	**Source (instrument or model)**	**Temporal resolution**
Displacement P1 to P7	Ground-Based InSAR	$1-6$ hours
Daily Mean Precipitation	Observation Based Interpolation	1 day
Daily Mean Air Temperature	Observation Based Interpolation	1 day
Daily Mean Snow Depth	Observation Based Interpolation	1 day
Freeze-Thaw Cycle Days	Derived from *Daily Mean Air Temperature*	1 day
Negative Degree Days	Derived from *Daily Mean Air Temperature*	1 day

The time series cover more than five years and the CPD divided the displacement dataset into 13 distinct windows (Fig. 6b).

**Fig. 7:**
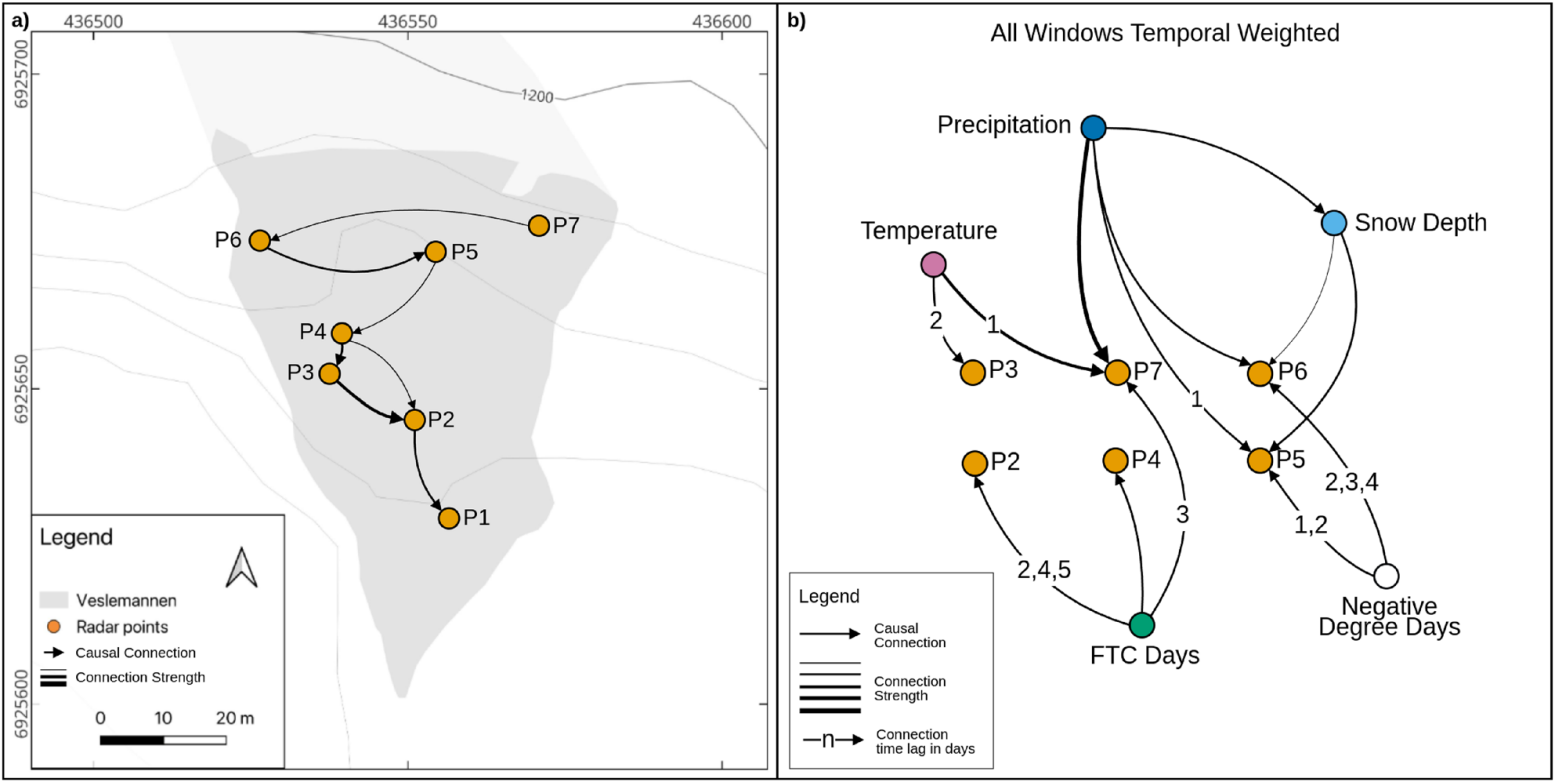
MWCD results for the case study site Veslemannen. **a**) Map showing the location of displacement measurement points on site with extracted weighted causal connections. **b**) Temporal Weighted causal graph including displacement and weather data for all time windows (Fig. 6b). Numbers refer to time lag in days. Map data provided by Kartverket (www.kartverket.no), map generated in QGIS 3.34 (www.qgis.org).

Here, Causal Discovery illuminates deformation dynamics across the unstable rock section Veslemannen. The causal graph showing the spatial causality (Fig. 7a) indicates that the slope deformation is driven from the lower parts of the deforming rock mass. The Temporal Weighted causal graph, exclusively presenting the influence of exogenous variables (Fig. 7b), shows that precipitation and the number of consecutive negative-degree days mainly affect the displacement in the lower part of the slope, either immediately or with a time lag between one and four days. This result is partly in accordance with Kristensen et al.^6^, who suggest that the deformation was controlled by precipitation and infiltration, i.e. snowmelt and rainfall, whereas slope displacement was assumed to be driven by the upper part of the rock mass. Our causal graph analysis further indicates that displacement in the upper part of the slope was mostly driven by FTCs and temperature, thus the transition from frozen ground conditions to thawing.

These results may aid in selecting predictor variables, including their lags and thresholds for displacement forecasting and early-warning systems. In addition, our analysis allows for identifying spatial strain distribution patterns, providing a better understanding of the internal geological processes.

## Discussion

Our research demonstrates the transformative potential of XAI-based Causal Discovery and its relevance for geoscientific applications. Based on the theory of causality, the method leverages the identification of causal relations between time series of monitoring data, whereas existing methods rely on correlation analysis and infer only apparent causal relations based on these correlations^2,6,34,37,8^, e.g.,. We show that MWCD is a powerful tool for identifying the importance of different displacement drivers on unstable rock slopes. The results for three different case study sites show the potential of the MWCD as an analytical tool of general validity, highlighting its applicability for the identification of different causal relations, including different spatial and temporal patterns. At our three case study sites, the types and time frames of available monitoring data differ greatly. However, the successful application of the MWCD demonstrates its versatility and reveals different driving mechanisms through the evolution of the instabilities. Our results are generally in accordance with previous expert-based analyses of the sites, but provide additionally a more unbiased and complete in-depth view of the system interactions and their changes over time. For example, our method identifies a hitherto undetected temporal shift from an indirect to a direct effect of precipitation and infiltration on the displacement at the Stampa rock section 4a. For the Preonzo case study site, the MWCD spatial causality discloses a considerable change in causal connections during the pre-failure acceleration from temperature and precipitation dominated effects to a gravitationally driven system. Lastly, at Veslemannen, our new method reveals that lower sections of the instability strongly influenced the deformation dynamics of the upper sections. Hence, drivers and regime shifts can be identified across all sites, albeit their differences in nature. This versatility of the method is of great practical relevance since no standardisation for monitoring networks exists and data availability varies greatly across sites. Thus, the MWCD gives us an unprecedented opportunity to analyse the long-term temporal evolution of influencing factors, identifying shifts in the significance of drivers and detecting significant short-term changes potentially leading to imminent failures. With increasing availability of displacement and environmental monitoring data, the demand for XAI-based solutions to effectively analyse these vast information collections has become more apparent. Using MWCD, the focus is not on a single main driver but instead on an ensemble of environmental factors potentially influencing the slope stability, and their evolution and interplay over time. This fills a knowledge gap in understanding rock slope instabilities, where potential drivers are commonly identified based on expert knowledge and correlation analyses^6–8^, e.g.,. The traditional approaches may miss potential destabilising factors, but above all, hardly allow for identifying and analysing temporal shifts and changes in the system.

Commonly used Deep Learning methods to extract temporal relations between time series, such as Long Short Term Memory (LSTM) based networks, are not capable of granting insight into the relations they extract from the data as their model weights do not reveal the variable relations. As such, these methods can be described as black boxes. Building the MWCD on XAI instead allows for the extraction of the causal system representation from the model after training. In the case of the MWCD, based on DAG-GNN, a matrix is learnt, representing the edges of the causal graph, including their directions^38^. Extracting the causal graph from the adjacency matrix as learnt by the XAI network, does not allow for a quantitative analysis in its current form and is limited to the qualitative interpretation we present. However, the Causal Discovery methods integrated into the MWCD framework have been applied to synthetic datasets in the past in order to demonstrate their effectiveness in reconstructing causal graphs when all assumptions to the input data are met^24,22,21^. A future direction could be to integrate an approach to learn edge functions that quantify cause and effect, since the directions of the effects have to be inferred manually in the presented approach. Thus, XAI-based Causal Discovery cannot be considered as replacement for traditional geological analyses but rather an additional tool providing complementary information that cannot be obtained through statistical analysis and thus allowing for a more holistic view. Causal graphs may be used for corroboration or discarding of assumptions on the slope behaviour, in addition to identifying still undiscovered drivers.

This analytical toolbox can further be enriched by slope stability analysis, for example using Limit Equilibrium Analysis or Finite Element Methods^11–13^. It is essential to note that the performance of XAI-based methods depends on a high-quality dataset, based on meticulous and comprehensive data collection. This includes interpolated data and data derived from a model, such as used for the OpenMeteo API, which must be verified to ensure it accurately represents the conditions at the site and the specific variables under examination. Furthermore, geoscientific domain knowledge is necessary for the evaluation of results, in order to avoid misinterpretations based on incorrectly oriented causal graph edges and unrealistic causal connections. Such misorientations and false causal links can be the result of too little variation in one time series, too few disparate time series or of including too many irrelevant variables^39^. Accordingly, our proposed method performs best in combination with domain knowledge allowing for analysis details where traditional methods fail to provide additional insights.

Besides, the results presented in this study aim at illustrating the potential of combining MWCD and explainable predictor networks on the selected case study sites. For detailed local studies, collecting high-quality in-situ data regarding external drivers, such as precipitation and temperature, is indispensable, as shown by our case studies. In general, more available data will produce causal graphs with higher confidence in the causal links and additional time series data may corroborate previously discovered causal links. The effect of different measurement errors on the model output is not considered in our analysis, but can further strengthen the interpretation of the model output.

Embracing XAI-based Causal Discovery methods in geoscience research holds ample potential for advancing our understanding of complex geological processes. These methods based on deep learning are able to uncover latent causal relationships, even in non-stationarity and complex interactions, under certain assumptions^40^. For example, the influence of latent variables that cannot be observed directly may be recovered if the variables have a widespread influence on observed variables^41,42^, e.g.,. This property holds the potential for deep insights into rock slope instabilities, where not all causal factors can be measured.

Additionally, the capability to handle high-dimensional data without prohibitive computation cost presents an advantage regarding high temporal resolution data, as high sample rates lead to more detailed insights into cause-and-effect relations between variables^43^. Graph Neural Networks, as used in the MWCD, are particularly well-suited for these time-series analysis tasks on potentially extensive datasets and can additionally enable anomaly detection and prediction^44^. The presented methodological toolbox is not restricted to surface displacement and meteorological data but can be applied to diverse time series datasets that may exhibit underlying causal relationships. Incorporating our innovative approach alongside traditional geological analyses holds the potential to revolutionise for example earthquake prediction, climate modelling, and natural disaster management. Unravelling the causal relationships between environmental factors will enable us to comprehend the underlying driving mechanisms better and develop improved anomaly detection and forecasting models. More specifically, causal graphs can be used to improve feature selection for prediction or automated susceptibility mapping, which leads to further refinement of existing machine learning-based approaches^45–47^, e.g.. By presenting a potential application of causal methods, we aim to inspire further exploration and refinement of these techniques in the scientific community.

## Methods

The proposed Multi-Window Causal Discovery (MWCD) computes causal graphs based on a multimodal input time series. The proposed algorithm (Fig. 1) is divided into the following steps:

- Data augmentation to derive negative-degree days and freeze-thaw cycles
- Pre-processing of displacement and environmental data
- Computing change points of displacement velocities, splitting the displacement time series into time windows
- Causal Discovery using a specified algorithm for each time window
- Computing the average occurrence rate of all edges, discarding edges that occur less in fewer than $30\%$ of the graphs to construct the weighted causal graph
- Adding temporally shifted versions of all time series
- Causal Discovery using a specified algorithm for every window of the time series using the time series augmented by shifting
- Computing the average occurrence rate of all edges, discarding edges that occur less in fewer than $30\%$ of the causal graphs over all windows or that are oriented backwards in time
- Combining information from the shifted and non-shifted causal graphs by annotating edges in the weighted causal graphs with the time lags from the temporal causal graphs

A technical implementation of the proposed workflow is provided in the cited GitHub repository.

### Data Augmentation and pre-processing

Best results were achieved using daily mean displacement velocities as inputs, rather than raw displacement values. Displacement data and exogenous variables such as meteorological and climate conditions are combined, augmented and pre-processed. Data augmentation consists of extending the dataset with information that would be lost in pre-processing due to rescaling. This concerns first and foremost the variable *negative-degree day* which indicates whether or not the mean temperature of a day was below $0^{\circ }\,C$. Since all variables are scaled independently to the range [0, 1], a loss of absolute value information is inevitable. In the case of negative-degree days however, this information is assumed to be important to the process understanding and is thus extracted prior to scaling. Additionally, we record how often a FTC was present and how many consecutive negative-degree days as well as FTCs were recorded.

Per window, the displacement time series is differentiated in order to force stationarity, which is assumed by the Causal Discovery algorithms and thus needed for acceptable results^24,22,21^.

Pre-processing results in a dataset with all variables formatted for use in a Deep Learning framework, i.e. with all time series values $t_i \forall i \in [0,n]$ in the interval $t_i \in [0,1]$.

### Change point detection

In order to create stationary time series for the Causal Discovery step we split the non-stationary displacement data into *time windows* using the Change Point Detection (CPD) algorithm Pruned Exact Linear Time (PELT). PELT is used with a Radial Base Function Kernel (RBF) with a penalty of 2. Additionally, a minimum window size of 30 samples is enforced. This minimum window size limits the number of windows and additionally enables the framework to identify meaningful causal relations over a minimum of 30 data points. The choice of minimum window size is however depending on the data set, especially on the number of features in the dataset. We recommend to choose a minimum window size strictly larger than the number of features.

We use the implementation from the freely available Python package *ruptures* (version 1.1.9, www.github.com/deepcharles/ruptures)^48^ and conducted a grid search to find an optimal penalty value. The resulting separations for the different penalties have been compared by-eye and the best separation with a penalty of 2 and minimum window width of 30 days selected for the automatic segmentation in the MWCD. For an in-depth discussion on the hyperparameter choice for PELT we refer to Haynes et al.^49^ and Truong et al.^50^.

Given that distribution shifts are detected and the time series split accordingly, we assume that the data in each window stems from the same distribution, enabling us to use Causal Discovery algorithms that demand stationary time series as input.

An additional consideration would be to automatically perform tests on the stationarity of the obtained segments, such as an Augmented Dickey Fuller test (ADF). The test outcome could be used to judge whether the time series segment is stationary. For all time series segments presented in this study, an ADF on the differentiated data resulted in a rejection of the Null-Hypothesis for a non-stationary time series with a significance level of $p<0.05$.

### Causal discovery

**Fig. 8:**
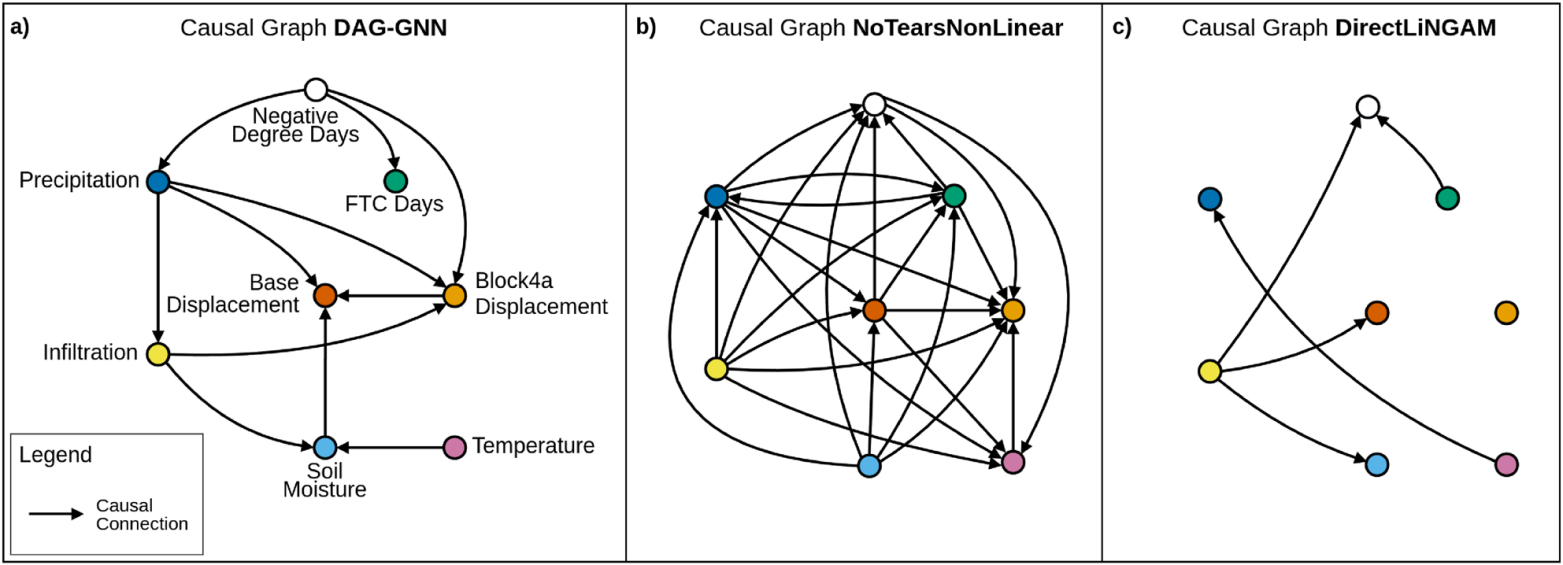
Representative example of Causal Graphs produced by the different Causal Discovery methods. Causal graph results using (**a**) DAG-GNN, (**b**) NoTearsNonLinear and (**c**) DirectLiNGAM.

The pre-processed data are used window-wise in combination with a Causal Discovery algorithm. We implemented the MWCD with three different deep learning-based algorithms: *DAG-GNN*^38^, *NoTearsNonLinear*^21^ and *DirectLiNGAM*^24^. DAG-GNN, NoTearsNonLinear and DirectLiNGAM have been used in their implementations as available in the freely available Python package for causal structure learning *gcastle* (version 1.0.3, www.pypi.org/project/gcastle/)^51^. Best results were achieved with DAG-GNN based on causal graph validation using domain knowledge. NoTearsNonLinear and DirectLiNGAM are extremely parameter sensitive and produced in most tests empty or fully-connected graphs. Moreover, DirectLiNGAM assumes a linear data generating process, which limits the identification of causal links to linear interactions between the causal features. We include the result of using the MWCD with DirectLiNGAM nonetheless to highlight the importance of allowing non-linear interactions between the observed variables. DAG-GNN (Fig. 8a), as opposed to NoTearsNonLinear (Fig. 8b) and DirectLiNGAM (Fig. 8c), consistently produced causal graphs with reasonable causal links, owing to its more robust structure and ability to account for non-linear relations.

However, as spurious correlations might lead to spurious causal links in the causal graph for all methods, we set a threshold of $30\%$ for edges in the summary graph^27^. This is in line with the threshold proposed by Yu et al.^38^. Hence, edges that occur in fewer than $30\%$ of all sections are not part of the summary graph.

All presented results are obtained using DAG-GNN during Causal Discovery. All three Causal Discovery methods are available for use in the MWCD made available through our repository that holds the complete implementation of the MWCD (https://github.com/Ci2Lab/MWCD).

The output of the Causal Discovery methods are matrices that encode the causal relations (graph edges) of the variables (graph nodes). The visualisations as provided in Figs. 3, 5 and 7 have been redrawn based on the matrix outputs. Simpler visualisations can be directly produced using open-source Python packages such as networkx^52^.

**Fig. 9:**
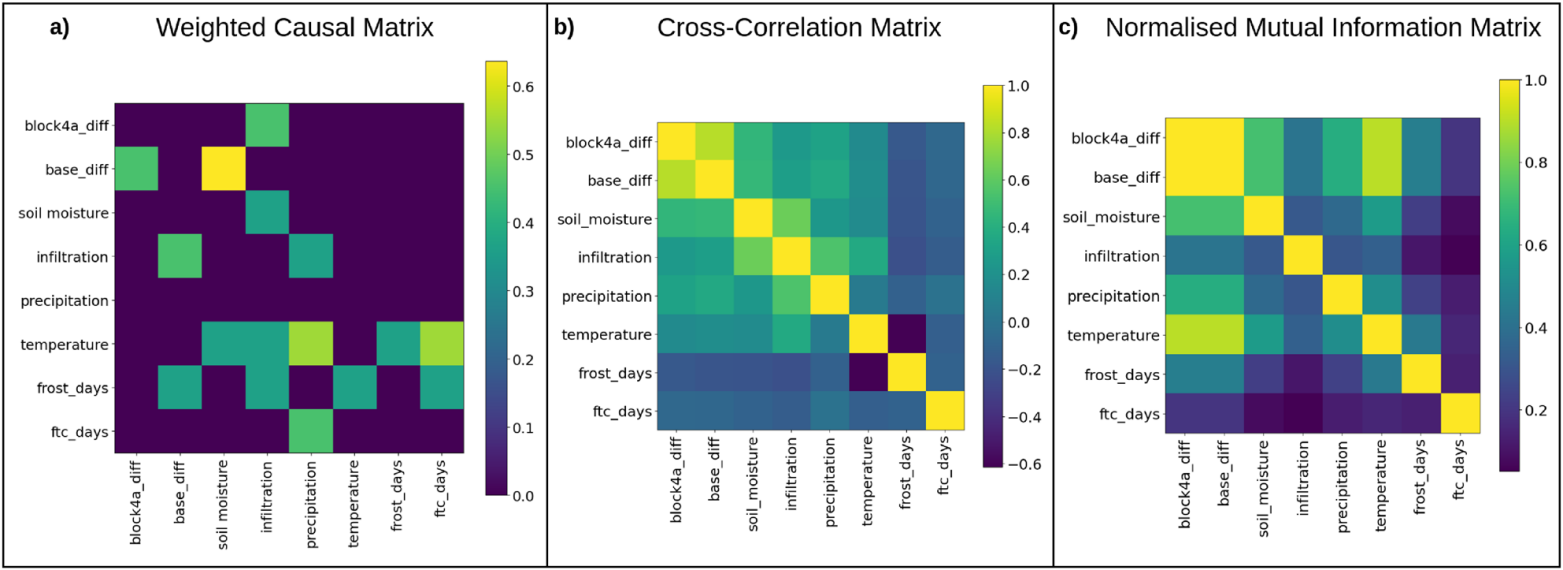
Differences between the Causal Matrix, the cross-correlation matrix and the normalised mutual information between the vairables for the Stampa case study site over all time windows. **a**) Causal Matrix as MWCD result encoding the average occurrence of the causal link over all segments with a threshold of $30\%$. Occurrence values are between 0 (never occurs) and 1 (always occurs). **b**) Cross-correlation matrix of all input time series. Matrix values range from $-1$ (perfectly negatively correlated) to 1 (perfectly positively correlated). The matrix is diagonally mirrored, encoding joint variation of the time series, with no difference in direction as opposed to the causal matrix in a). **c**) Normalised Mutual information scores for all variable pairs. The matrix is diagonally mirrored, indicating how much information the observation of one variable reveals about another variable. The normalised values range from 1 (maximum of information gain) to 0 (independence of the variables).

A comparison with a cross-correlation matrix and the pairwise normalised mutual information scores reveals the differences between a Causal Discovery and statistical analysis (Fig. 9).

While Causal Discovery as implemented in the MWCD results in a Causal Matrix encoding the cause and effect relations between the features (Fig. 9a), the cross-correlation matrix identifies features that vary together, without an indication of a cause and effect relation in the variation (Fig. 9b). Furthermore, the correlation coefficients only measure linear dependence between the variables. The Normalised Mutual Information scores for all variable pairs, a measure for statistical dependence not limited to linear dependencies here normalised to a value between 0 and 1, quantifies how much information about one variable can be obtained from observing one of the other variables^53,54^. As such, the three matrices present complementary information, strength of causal links between variables, linear correlation and non-linear strength of variable connections, illustrating the effectiveness of complementing a statistical analysis with Causal Discovery.

#### Software and hardware requirements

All results presented have been computed using Python 3.11.4 (www.python.org) in combination with *pandas* (version 1.5.3, www.pandas.pydata.org), *numpy* (version 1.23.5, www.numpy.org) and *pytorch* (version 2.0.1, www.pytorch.org) on a Laptop with a 16 core AMD Ryzen 9 6900hx processor with 32GB memory and a NVIDIA GeForce GTX 3070 Ti Graphic Processing Unit (GPU) with 8GB memory using CUDA 12.7, as the MWCD allows for using a dedicated GPU to speed up the processing. The average computation time for the Stampa case study site with the indicated settings over ten runs is $989.3 \pm 46$ seconds.

#### Data resolution and measurement errors

The spatial and temporal resolution of the data have a direct impact on the interpretability of the causal graph. All input data should have the same sampling interval and the minimum discoverable time lag will be one full sampling interval. Spatial resolution on the other hand does not have a direct effect on the method, but the distribution of measurement points for displacement is decisive for the interpretation of the causal graphs, i.e. measurements at more locations will lead to a higher spatial resolution of the causal graph, but do not affect the overall output of the MWCD.

The accuracy of the observations directly affects the accuracy of the causal graphs the MWCD produces. While systematic measurement errors are assumed to have a negligible effect on the MWCD output due to the normalisation of all inputs, noise on the observations, i.e. random noise measurement errors, can influence the causal graph accuracy. This is generally true when few samples are used as input to a causal discovery method, while the output accuracy is less affected when a large number of samples is used as input^55^. Temporally aggregating samples with a high sampling rate over a given period further reduces the effect of measurement errors on the accuracy of the model output^16^.

## Data Availability

The datasets generated and/or analysed during the current study are available in the MWCD repository for rock section 4a under https://github.com/Ci2Lab/MWCD, while data for the Preonzo and Veslemannen sites is available under https://doi.org/10.14459/2023mp1688868.
